# The Effect of Photodynamic Therapy on the Smear Layer Removal: a Scanning Electron Microscopic Study

**DOI:** 10.30476/DENTJODS.2020.85208.1118

**Published:** 2021-09

**Authors:** Negin Ghasemi, Zahra Sadat Torabi

**Affiliations:** 1 Dept. of Endodontics, Dental and Periodontal Research Center, Dental Faculty, Tabriz University of Medical Sciences, Tabriz, Iran; 2 Dept. of Oral and Maxillofacial Surgery, School of Dentistry, Shahid Beheshti University of Medical Sciences, Tehran, Iran

**Keywords:** Photochemotherapy, Root Canal Therapy, Smear layer

## Abstract

**Statement of the Problem:**

The efficacy of photodynamic therapy (PDT) in smear layer removal compared to the currently accepted protocol is not well established.

**Purpose:**

This study aims to evaluate the effect of PDT on smear layer removal from human root canal compared to combined use of irrigation solutions including sodium hypochlorite (NaOCl) and ethylene diamine tetra acetic acid (EDTA) by scanning electron microscopy (SEM).

**Materials and Method:**

In this *in vitro* study, straight roots from 48 extracted human maxillary incisors and canines were selected and decoronated. Instrumentation was completed with RaCe rotary system and normal saline irrigation between files. Then roots were randomly divided into 3 groups (n=16). Group 1 was the control group to confirm smear layer formation. In the group 2, the canals were irrigated with 2ml of 2.5% NaOCl solution for 10 minutes and 2ml of 17% EDTA solution for 1 minute. In the group 3, PDT with methylene blue and diode laser (625nm, 150mW, for 5minutes) was the final procedure for smear layer removal. All the specimens were sectioned into two halves, gold coated, and analyzed under SEM. The smear layer in the coronal, middle, and apical thirds, were evaluated and scored by two examiners independently. Statistical analysis was done using Chi-square test.

**Results:**

It was observed that the NaOCl+EDTA removed the smear layer significantly better than PDT in the coronal and apical thirds (*p*< 0.05) whereas PDT was unable to remove the smear layer in none of the root areas. At the apical thirds, there was no significant difference between NaOCl+EDTA and PDT (*p*< 0.05). Both procedures were unable to remove smear layer from radicular dentine of this area.

**Conclusion:**

According to the results of this *in vitro* study, the use of PDT alone is not recommended to remove smear layer. The combined application of NaOCl and EDTA is ineffective in removing smear layer of apical third, despite its efficacy on the coronal and middle regions.

## Introduction

Smear layer is the inevitable result of mechanical preparation of the root canal that includes pulp tissue remnants, dentin, and necrotic debris. In general, the smear layer consists of organic and nonorganic components and can harbor bacteria [ 1 ]. Despite differences in earlier opinions on the need to remove the smear layer, evidence from systematic review shows that when this layer is not removed, fluid seal after canal obturation is negatively affected [ 2 ]. As a result, trying to remove this layer, especially in infectious cases is prudent for canal disinfection [ 3 ]. The presence of this layer blocks the orifices of dentinal tubules, which impedes the adhesion of sealers and biomaterials like MTA to the canal’s wall and reduces the resulting seal. It is considered as a significant barrier for effective performance of these materials [ 2 , 4 - 5 ]. 

Today, the standard protocol for the smear layer removal is combined use of sodium hypochlorite (NaOCl) and ethylene diamine tetra acetic acid (EDTA). NaOCl is the most common irrigating solution used in root canal therapy, which is proteolytic with proper antimicrobial properties. However, it is not able to remove the nonorganic parts of the smear layer [ 6 ]. Similar problem exists in other solutions and since the smear layer is a complex and resistant combination, no solutions have been able to remove both organic and nonorganic parts yet [ 7 ]. EDTA is a chelating agent that solves the nonorganic part of the smear layer. However, this material, if not washed on time from the canal, would cause dentin erosion [ 8 ]. Nevertheless, the combined use of these two solutions is recommended for effective removal of all components of the smear layer as the final step in cleaning the canal. 

In addition to irrigation solutions, lasers are newer methods for clearing the canal. Preliminary results of using high-power lasers in cleaning the canal was very promising [ 9 ]. Lasers penetrate inaccessible canal areas, and are considered for their strong antibacterial effects in endodontics [ 10 ]. In addition, lasers affect the dentinal tubules orifice and melt and occlude tubules, and hence, result in tubules’ seals. In particular, this effect is more prominently observed in one third of the apical part, which is important for reducing microleakage [ 11 ]. However, high temperature and risk of complications, such as damage to periapical tissues, resorption, and ankylosis are the disadvantages of using high power lasers [ 10 ].

Photodynamic therapy (PDT) is a new method that evokes a nontoxic photosensitizer using low level lasers in the presence of oxygen and acts by generating free radicals [ 12 ]. The benefits of this method includes no damage in the periapical tissues and eliminating *Enterococcus faecalis* bacteria that is frequently associated with root canal therapy failure causing refractory apical periodontitis [ 13 ]. PDT has less cytotoxicity compared to other common irrigation solutions [ 14 ]. The effectiveness of PDT is based on a photochemical process with high selectivity and is not associated with minor increase (>0.5°C) in temperature [ 15 ]. As suggested, minor increase in temperature caused by the use of PDT can cause fibrogenesis and neo-angiogenesis and accelerates healing [ 16 ].

Regarding the anti-microbial and disinfecting effects of PDT, numerous researches have been conducted and researchers have concluded through systematic review that despite the lack of clinical evidence, PDT may be used as adjunct treatment for cleaning the canal [ 17 ]. However, there is no information about the effectiveness of this method in removal of the smear layer, compared to other methods of smear layer removal. The present study aimed to compare the efficiency of PDT with standard protocol in removal of the smear layer from the canal of extracted teeth in coronal, middle, and apical areas of the canal with use of scanning electron microscopy (SEM). 

## Materials and Method

### Collection and Preparation of the samples

In this *in vitro* study, 48 freshly extracted human maxillary incisors and canines were collected and stored in 0.5% Chloramine-T. Roots were fully developed, straight without previous root canal therapy and free of caries. Presence of single root canal was confirmed radiographically. All teeth were decoronated with diamond disc (SP 1600 Microtome, Leica, Nu Block, Germany) under water coolant to leave 12 mm of root length. To obtain the working length of each root a 15# k-file (Dentsply Maillefer, Ballaigues, Switzerland) was placed into the canals. Working length was set to be 1 mm shorter than anatomic apex. To control the working length and provide patency a 10# k file was passed through the apical foramina before initiation of instrumentation. Then sticky wax was used to seal apical foramina to avoid immediate evacuation of solutions. Instrumentation was completed using the crown-down technique with RaCe rotary system (FKG Dentaire, La-Chaux-de Fonds, Switzerland) as described: #40/0.10 and # 35/0.08 for the coronal third, #30/0.06 for the middle third and #25/0.06 for preparation up to the working length. Canals were irrigated with normal saline during instrumentation using a 27-gauge needle placed 1mm shorter than working length. 

### Smear layer removal procedure

For smear layer removal, all specimens were randomly divided into three groups of 16 roots according to the type of procedure for the final smear layer removal. In the group1 (control group), no additional procedure for smear layer removal was done for this group.

 In the group 2 (NaOCl+ EDTA), 2ml of 2.5% NaOCl was used for 10 minutes. Subsequently, canals were flushed with 10 ml of sterile distilled water. Then 2ml of 17% EDTA (Pulpdent Corporation, Watertown, MA, USA) was used for 1 minute followed by 10 mL of distilled water as final flush to avoid prolonged effect of EDTA. The root canals were dried with sterile paper points (Ariadent, Iran).

In the group 3(PDT), all individual roots were mounted on a sterile sponge. The procedure was done in a room with minimum light. A total of 1 mL of 0.01% methylene blue (MB) was used as photosensitizer. MB was filter-sterilized immediately before procedure and filled into root canals with a 27-gauge needle for 5 minutes. The canals were dried with paper cones subsequently. The irradiation was done using a diode laser (Fotoson CMS Dental, Denmark) as light source for 2.5 minutes. Irradiation was stopped for 2.5 minutes followed by another irradiation for 2.5 minutes. Laser was used at 625nm wavelength, output power of 150 mW, power density of 1.4W/cm^2^, spot size of 0.07cm^2^, energy density of 214.28 J/cm^2^, and energy of 15 J in continuous mode. 

To achieve a 360-degree uniform irradiation, laser was coupled with a flexible optical fiber with a diameter of 200 micrometers and 3 % taper. Fiber was fully inserted into canal, so the tip was placed 2 mm shorter than working length. Finally, canals were irrigated with sterile distilled water using 27-gauge sterile needles for each canal.

### SEM preparation and analysis

All samples were analyzed with SEM. Two parallel longitudinal grooves were cut on each root with diamond disc and then the roots were split into 2 halves using bi-beveled chisel and mallet to prevent inner surface manipulation. one section of each root that had a better view of the apical region was dehydrated in graded alcohol series for 24 hours (70%–100%), then coated with gold sputtering, and finally, analyzed with SEM (MRIA3-FEG-SEM) connected to EDX (EMAX7000 Type S; Czech). SEM photomicrographs were taken from 3 thirds of the canal: coronal, middle and apical. Observations were carried out under 2500× magnification. The photomicrographs were analyzed by two specialists in endodontics that were blind to study groups. Disagreements were solved by consensus. Smear layer was scored according to Torabinejad *et al*. [ 18 ]. In this scale, score 1 indicated no smear layer and clean dentinal tubules, score 2 indicated moderate smear layer (no smear layer present on the surface of root canal, but tubules contaminated with debris), and score 3 indicated heavy smear layer (the root canal surface and the tubules covered with smear layer). 

### Statistical analysis

Data was tabulated and analyzed using SPSS software (SPSS version 18.0, SPSS, Chicago, IL, USA). To determine the conformance among the two examiners, Kappa agreement coefficient was used. To check the difference between percentages of the obtained scores in coronal, middle, and apical thirds, Chi-square test was used. Significance of tests was set at 0.05. 

## Results

The smear layer removal rate was monitored by two observers. According to the results, Kappa agreement coefficient calculated between these measures showed complete agreement between the results of two observers (K = 0.99). 

**Figure 1:**
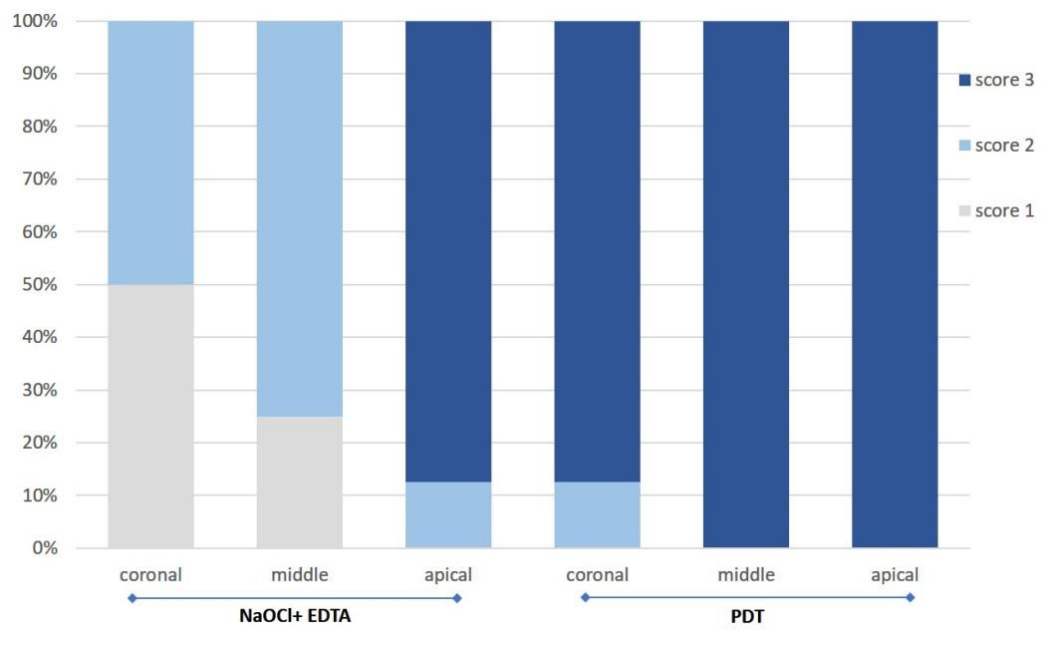
The distribution of smear layer scores

**Figure 2:**
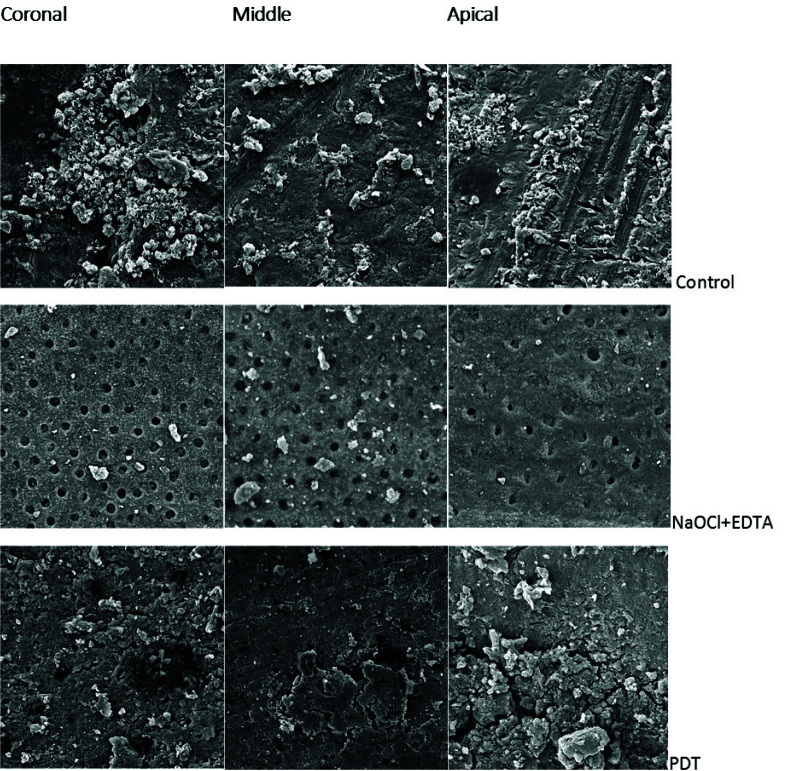
SEM photomicrographs of each group

The results obtained from the smear layer scores are presented in Table 1. The distribution of smear layer scores is presented in Figure 1. Figure 2 shows representative SEM photomicrographs of each group. According to the chi-square test results, the difference between the obtained scores were significant in coronal and middle sections (*p*< 0.001) and non-significant in apical section (p= 0.48> 0.05). All of the photomicrographs obtained from control group (group 1) revealed a thick smear layer in coronal, middle, and apical thirds. The difference between the percentages of the obtained scores in coronal, middle and apical thirds, with Chi-square test indicates the highest rate of complete removal of the smear layer (score 1) in NaOCl+ EDTA group (group 2) and coronal sections (50%). Intermediate removal of the smear layer (score 2) was highest in NaOCl+ EDTA group (group 2) and middle thirds (75%). Moreover, in PDT group, the smear layer that completely remained (score 3) was observed in all thirds of the canals (100%).

**Table1:** The smear layer score frequency for different procedures

Dental sections	Groups	Scores			*p* Value
		1 (%)	2 (%)	3 (%)	
Coronal	EDTA	8(50%)	8(50%)	0	<0.001
	PDT	0	2(12.5%)	14(87.5%)	
Midline	EDTA	4(25%)	12(75%)	0	<0.001
	PDT	0	0	16(100%)	
Apical	EDTA	0	2(12.5%)	14(87.5%)	0.48
	PDT	0	0	16(100%)	

## Discussion

The aim of this study was to assess the efficiency of PDT in removing the smear layer from the root canal surface in different sections, compared with the conventional protocol (NaOCl and EDTA).The results showed that PDT could not remove the smear layer in coronal, middle, and apical thirds of the root canal. 

Recently, a systematic review identified that despite scarce number of studies, clinical evidence supports the smear layer removal [ 19 ]. To remove the smear layer, different irrigants are suggested, including NaOCl, EDTA, maleic acid, citric acid, and MTAD (mixture of Doxycycline, citric acid and a detergent), as well as using lasers or a combination of these materials [ 7 - 8 , 20 ]. Application of 2.5% NaOCl and 17% EDTA is one of the currently approved protocols by researchers [ 7 , 21 ]. Thus, in this study, this protocol was used to be compared for the effect of PDT. EDTA is used in different durations. In this study, following similar studies, EDTA was used for 1 minute. According to the study by Teixeira *et al*. [ 21 ], the application of EDTA for 1, 3, and 5 minutes has presented similar effectiveness. Short time application of this material would decrease the adverse effects of the prolonged presence of this material on dentin wall [ 22 ].

The use of lasers for final disinfection of the canal is controversial concerning the considerable thermal effects of application of lasers. In addition to the destructive effects of increased temperature on apical tissues, Matsouka *et al*. [ 23 ] reported formation of crack on dentin walls. In this study, the new approach of PDT was evaluated that has shown promising antimicrobial effects in laboratory studies [ 13 ]. This method does not have the common complications of lasers considering the light source with lower power and the cleaning process, which is based on the photochemical phenomenon [ 15 ]. Adjustment of power, wavelength, and duration of laser were selected regarding the previous studies [ 24 ].

In the current study, similar to a number of previous studies, MB was used as photosensitizer for PDT [ 25 ]. Previous studies rejected cell toxicity of this material. The molecular properties of this material enable it to penetrate in gram negative bacteria by porin-protein canals of extracellular membrane [ 15 ].

In this study, SEM was used to evaluate the results. The high magnification power of SEM enabled accurate observation of dentine tubules and is thus widely used in studies on smear layer [ 3 , 8 ]. The findings of this study showed that PDT could not be used for simultaneous removal of smear layer and cleaning the canal from resistant bacteria. The conventional combinational method has significantly higher power in removal of smear layer, compared to PDT. To our knowledge, there are no studies on the effect of PDT on smear layer removal; thus, we could not compare the results. The results of the present study indicated that, despite the antimicrobial effects shown in previous studies, this method should not be used alone or as an alternative to current protocols. This is because the inability to remove the smear layer limits the anti-microbial effects of PDT on canal’s space. Formerly, studies recommended this treatment as adjunct method. Garcez *et al*. [ 26 ] used NaOCl and EDTA in their study before employment of PDT and concluded that adjunctive use significantly improved PDT outcome. This result is consistent with a systematic review by Cherpa *et al*. [ 17 ]

The efficiency of using NaOCl and EDTA on smear layer removal in coronal and middle sections was confirmed by the present study and previous studies [ 21 ]. However, using these two materials may also have adverse effects. Low biocompatibility of these two materials is one of the adverse effects, especially if they reach beyond the apex [ 27 - 28 ]. Otherwise, if EDTA is not washed well from the canal, it causes erosion of radicular dentin wall and has adverse effects on the microhardness of dentin wall [ 22 ].

 In apical section, there was no significant different in the rate of the smear layer removal by two study methods and none of the two methods were able to eliminate the smear layer from the canal in this section. The inability of NaOCl and EDTA to remove the smear layer completely from the one third of the apical section, observed in this study, is in agreement with the study of Rathakrishnan *et al*. [ 3 ]. The conventional method, despite the success in removing the smear layer in the coronal and middle sections, is not effective on smear layer removal of the apical third, which is attributed to the vapor lock effect. The vapor lock is formed because the end of the canal has a closed end and is tighter, which prevents the circulation of irrigant solutions [ 7 , 21 ]. Gulabivala *et al*. [ 29 ] also explained that it could be due to lack of cleansing the apical region with the lack of penetration of the needle tip and the creation of a stagnation plane beyond the tip. Studies have provided various solutions to this problem and one method to improve the performance of irrigants in the apical third is agitation with ultrasonic heads, which had good results [ 7 ].

## Conclusion

Considering the limitations of the current study, the use of PDT is not recommended for removing the smear layer. PDT can be used as an adjunctive protocol for disinfection after removal of the smear layer by the conventional protocol.
